# Supercritical Antisolvent Technique for the Production of Breathable Naringin Powder

**DOI:** 10.3390/pharmaceutics14081623

**Published:** 2022-08-03

**Authors:** Renata Adami, Paola Russo, Chiara Amante, Chiara De Soricellis, Giovanna Della Porta, Ernesto Reverchon, Pasquale Del Gaudio

**Affiliations:** 1 Department of Physics E. Caianiello, University of Salerno, Via Giovanni Paolo II, 132, 84084 Fisciano, SA, Italy; 2 Department of Pharmacy, University of Salerno, Via Giovanni Paolo II, 132, 84084 Fisciano, SA, Italy; paorusso@unisa.it (P.R.); camante@unisa.it (C.A.); cdesoricellis@unisa.it (C.D.S.); 3 Department of Medicine, Surgery and Odontoiatry, Scuola Medica Salernitana, University of Salerno, Via Salvatore Allende, 1, 84081 Baronissi, SA, Italy; gdellaporta@unisa.it; 4 Department of Industrial Engineering, University of Salerno, Via Giovanni Paolo II, 132, 84084 Fisciano, SA, Italy; ereverchon@unisa.it

**Keywords:** supercritical antisolvent extraction, naringin, microparticles, long COVID, inhalation therapy

## Abstract

Flavonoids are polyphenolic compounds largely present in fruits and vegetables possessing antioxidant properties, anti-inflammatory and antibacterial activities. Their use in clinical practice is very poor due to their low bioavailability, susceptibility to oxidation and degradation. Moreover, their slight solubility in biological fluids and a consequent low dissolution rate leads to an irregular absorption from solid dosage forms, even though, anti-inflammatory formulations could be used as support for several disease treatment, i.e. the COVID-19 syndrome. To improve flavonoid bioavailability particle size of the powder can be reduced to make it breathable and to promote the absorption in the lung tissues. Supercritical fluid based antisolvent technique has been used to produce naringin particles, with size, shape and density as well as free flowing properties able to fit inhalation needs. The dried particles are produced with the removal of the solvent at lower temperatures compared to the most used traditional micronization processes, such as spray drying. The best breathable fraction for naringin particles is obtained for particles with a d_50_~7 µm manufactured at 35 °C-150 bar and at 60 °C-130 bar, corresponding to 32.6% and 36.7% respectively. The powder is produced using a high CO_2_ molar fraction (0.99) that assure a better removal of the solvent. NuLi-1 cell line of immortalised bronchial epithelial cells adopted to evaluate powder cytotoxicity indicated after 24 h absence of toxicity at concentration of 25 µM.

## 1. Introduction

The enhancement of the body antioxidant defenses through the diet or through a pharmacological uptake is a very interesting approach in the modern medicine. Natural antioxidants, flavonoids, polyphenolic compounds largely present in fruits and vegetables have anti-inflammatory, antibacterial activities and antioxidant properties [1,2,3,4]. Despite their efficacy, the use of flavonoids in clinical practice is very poor mainly due to their susceptibility to oxidation, degradation at gastric pH, very slight solubility in biological fluids and very low dissolution rate, leading to an irregular absorption when administered by an oral solid dosage form [5]. Therefore, administration of flavonoids by an inhalable formulation could lead to high local concentration of the antioxidant in the respiratory tissues avoiding gastro-intestinal degradation. Such formulations in form of nebulizer or dry powder inhaler (DPI) could also enhance systemic absorption due to lungs high surface absorption area and vascularization [6] and could prove very helpful in managing environmental threats directly addressed to the respiratory system.

In the lungs there is a physiologic oxidant-antioxidant balance that easily can move in favour of oxidants in case of exposure to pollution, smoke, chemical and biological agents present in the inhaled air. This leads to lung inflammation or even injury [7]. The upper respiratory tract has the role to protect the respiratory system from the agents in the inhaled air and the inflammation related to tissue oxidant stress produce acute and chronic inflammatory diseases [8,9,10,11]. 

Furthermore, the imbalance in the redox state in favour of oxidant conditions in the lung is one of the main issues in respiratory viral infections, linked to inflammation and subsequent tissue damage. Recent evidence indicates that oxidation also plays a crucial role in COVID infection and its long-term effects. Antioxidants, as additional protective molecules in the tissues of both upper and lower respiratory tracts, can reduce pulmonary inflammation and tissue damage, providing a useful tool for containing COVID infection and avoiding lung injuries in the so-called long COVID syndrome [9,12,13,14]. It has been already proved that several antioxidants loaded inhalable formulations can reduce the oxidant stress of lung and nasal mucosa due to the controlled delivery of flavonoids and phenolic compounds [15,16,17]. Nevertheless, the use of a dry powder formulation can also improve the antioxidant biomolecule physicochemical and microbiological stability, thanks to a higher drug concentration at the deposition site [18,19,20].

Proper particle properties are necessary to achieve lung airways deposition, and to avoid aggregation or poor powder flowability during formulation manufacturing. A number of attempts to improve inhalable powders performance, including the modification of density, particle morphology, surface and porosity, as well as the blending of the active drug powder with inert carriers, have been reported [21,22,23].

Naringin is the main flavonoid present in several fruits of the Citrus family, mainly in grapefruit (*Citrus paradisi*), and is one of the main active components of Chinese herbal medicines [24]. It has antioxidant, anti-inflammatory, anti-apoptotic, anti-ulcer, anti-osteoporotic and anti-carcinogenic properties [25] and is absorbed by the gut, therefore it has a low bioavailability through oral administration. Recent studies report that naringin is among the flavonoids promising to be effectively used in therapies against COVID. It easily binds to the COVID receptor and, due to its anti-inflammatory properties, it is able to inhibit the expression of some (inflammatory) cytokines induced by the infection [26,27,28].

Naringin has been micronized using spray drying technique, obtaining amorphous powders with controlled shape, breathable size and tap density suitable for airways deposition [29]. Supercritical fluid processes have been proposed to produce particles of micro and nanometric size [30], composite microparticles [31,32,33,34,35], and, in particular, microparticles for aerosol delivery [36,37,38,39,40,41]. Supercritical Antisolvent or Supercritical Antisolvent Extraction techniques, in which supercritical CO_2_ is used to remove the solvent from a solution in order to obtain precipitation of a powder, have been used for the production of antioxidant microparticles [42,43], mainly loaded in PVP microparticles [34] and for fractionation of antioxidants from natural matrices [44,45,46,47,48,49,50].

In this work supercritical antisolvent technique is proposed to manufacture inhalable powder of naringin, used as antioxidant active ingredient model, with the proper size, shape and density as well as free flowing properties to reach the deep airways. The aim of the work has been to obtain dried particles of pure naringin, without adding any excipient, effective for inhalation application. The powder is obtained using a solvent removal technology at lower temperature compared to the most used traditional micronization processes, for example spray drying, thus increasing biomolecule stability. Powder mean size and morphology coupled with solid state characteristics were monitored by laser granulometer, electron microscopy and XRay analyses coupled with breathable experiments. Microparticles cytotoxicity was also investigated by MTT assay on NuLi-1 cell line of immortalised bronchial epithelial cells in order to verify the biocompatibility of the powder after supercritical CO_2_ treatment.

## 2. Materials and Methods

### 2.1. Materials

Pure naringin and ethanol (EtOH, 99.8%) were supplied by Sigma-Aldrich (Milan, Italy); carbon dioxide (CO_2_, purity 99%) was purchased from SON (Naples, Italy).

### 2.2. Micronization Method

**Figure 1:**
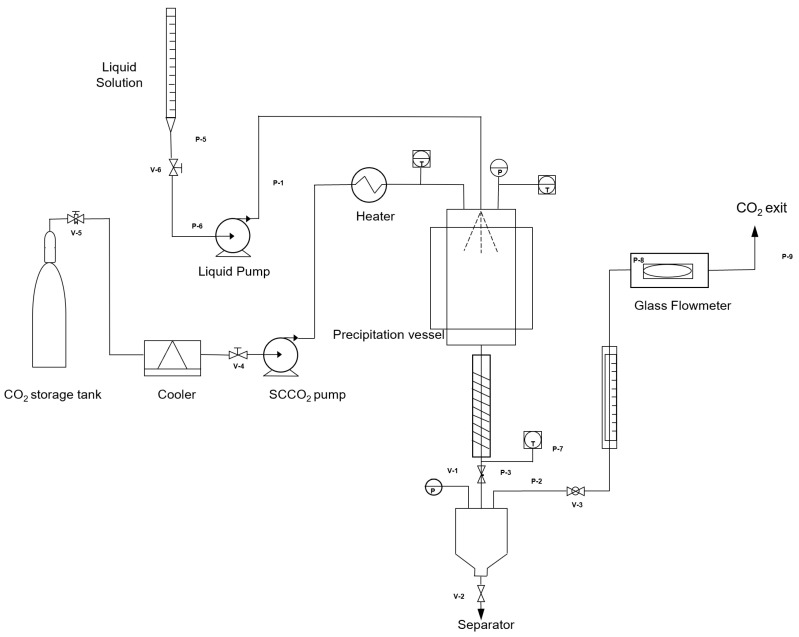
Schematic representation of SAE apparatus.

Micronization of naringin was performed by supercritical antisolvent extraction (SAE) technique. A membrane high-pressure pump (LEWA, mod. LDB1 M210S) was used to deliver CO_2_ and a HPLC pump (Gilson, mod. 305) was used to deliver the liquid solution (extract from the C18 cartridge). The precipitator was a stainless-steel vessel (*V* = 0.4 L, i.d. = 50 mm). A 180 µm nozzle on the top of the precipitator allowed the injection of the liquid solution and a stainless-steel filter having a porosity of 1 µm, located at the bottom, allowed the collection of the powder material. A separator, located downstream from the pressure reduction valve, and operating at 3 MPa, was used to recover the liquid solvent. Figure 1 shows a schematic representation of the equipment used for the experiments. Further details can be found elsewhere [45,50].

To perform SAE experiments, CO_2_ was first pumped to the precipitator at a fixed temperature until the desired condition of pressure were reached; then, the pressure was regulated by a micrometric valve located between the precipitator and the separator. When a constant flow rate of CO_2_ was established, pure solvent was sent through the nozzle to the precipitator until steady state conditions for solvent and antisolvent system were reached. At this point, the delivery of the ethanolic solution started. The fast extraction of the solvent by SC-CO_2_ produced the precipitation of the solute. At the end of the ethanolic solution delivery, the precipitator was purged with pure SC-CO_2_ under the process conditions, to wash away residual solvent solubilized in the supercritical antisolvent. If the final purge with pure SC-CO_2_ was not performed, the solvent contained in the antisolvent condensed during depressurization, solubilizing or modifying the precipitate. Finally, the pressure of the precipitation vessel was decreased, and the powder obtained was collected. The ethanol could be recovered in the separator operated at 30 bar.

### 2.3. Particle Morphology and Size Distribution

The morphology of micronized naringin was analyzed by a Field Emission Scanning Electron Microscope (FESEM, mod. LEO 1525, Carl Zeiss SMT AG, Oberkochen, Germany). Powders were dispersed on a carbon tab previously stuck to an aluminum stub (Agar Scientific, Stansted, UK). Samples were coated with gold-palladium (layer thickness 250 Å) using a sputter coater (mod. 108 A, Agar Scientific, Stansted, UK). At least 20 SEM images were taken for each batch to verify the powder uniformity.

Particle size (PS) and particle size distribution (PSD) were measured by a laser light scattering granulometer (LLS) equipped with a liquid module (LS 13-320 Beckman Coulter Inc., FL, USA); the LLS uses a laser operating diode at 5.0 mW and 750 nm, which measures the hydrodynamic diameter of the particles in the range of 0.5–1000 µm. Untreated and micronized naringin particles were dispersed in dichloromethane and sonicated for 2 min. Then, a few drops of the suspension were poured into a small cell in order to obtain an obscuration between 8% and 12%. The function of the distribution of the particle size was predicted by the instrument software, using the Fraunhofer method. The analyses for each sample were performed in triplicate and the results are expressed as 10%, 50%, and 90% of the distribution of the particles size (d10, d50, and d90, respectively).

### 2.4. Powder Density

Powder density was measured using an automatic gas ultra-pycnometer (1000, Quantachrome GmbH & Co. KG, Odelzhausen, Germany), equipped with cells and spheres having different sizes for different applications. The gas used was helium, at a pressure of about 19 psi. Before each measurement, the instrument was calibrated using a cell of 10 cm^3^ and a sphere of 7.0699 cm^3^.

### 2.5. X-ray Powder Diffraction Analyses (XRPD)

Crystallographic analysis of the different samples was performed using an X-ray diffractometer (mod. D2 PHASER 2nd Gen, Bruker AXS, Inc., Madison, WI, USA) with a Cu sealed tube source. Samples were placed in the holder and flattened with a glass slide to ensure a good surface texture. The measuring conditions were as follows: Ni-filtered CuKα radiation, λ = 1.54 A, and 2θ angle range of 5–70° with a scan rate of 3 s/step and a step size of 0.02°.

### 2.6. Particle Breathability Analyses

The in vitro deposition of micronized naringin was evaluated using a glass impinger at a single stage (SSGI, European Pharmacopoeia 6.0 instrument, Copley Ltd. Scientific, Mothingam, UK).

A water/ethanol 9/1 *v*/*v* mixture was introduced in the upper (7 mL) and lower (30 mL) stages of the SSGI. Hard gelatin capsules (size 2) were filled with 20.0 ± 0.5 mg of naringin powders and introduced into the Turbospin. The vacuum pump was operated at a flow rate of 60 L/min for 5 s. Each deposition experiment was carried out on 10 capsules and repeated in triplicate. A UV spectrometer (UV/Vis spectrometer Lambda 25, Perkin-Elmer instruments, Waltham, MA, USA) was used at a wavelength of 283 nm to quantify the naringin deposited into the upper and the lower stages of the impinger. The analytic method was validated using standard solutions of naringin in the range of 70–400 mg/L. The emitted dose (ED) was gravimetrically determined and expressed as the percentage of the powder at the exit of the apparatus vs. the amount of powder introduced into the capsule. The ratio between the naringin recovered from the SSGI and the naringin emitted from the device was the recovered dose. The fine particle fraction (FPF) was defined as the percentage of the ratio of the naringin recovered from the lower stage of SSGI vs. the total loaded dose.

### 2.7. In Vitro Permeation Study

Permeation assays were performed by means of Franz-type vertical diffusion cells (Hanson research corporation, Chatsworth, CA, USA). The cell system temperature was kept constant at 37 °C throughout the experiment by recirculating water from a thermostatically controlled bath. Continuous stirring (170 rpm) was provided by a Teflon-coated stirring bar placed in the receptor compartment. A nitrocellulose membrane (pore size, 0.45 µm) previously set with phosphate buffer (0.05 M, pH 7.4) was applied between donor and receptor compartments (permeation area 1.77 cm^2^). The receptor compartment was filled with the same phosphate buffer. About 25 mg of the selected powder, precisely weighed, was uniformly spread on the membrane surface. Samples of 100 µL were removed at defined time intervals (5, 10, 15, 20, 30, 45, 60 min), replacing the same volume of warmed buffer in the receptor. The aliquots were analyzed for naringin content by UV as reported above. The amount of the naringin permeated per area (Q) for each time interval was calculated by means of the following equation:

(1) $$Q\left(\frac{\mu g}{cm^{2}}\right)=\frac{V_{R}\cdot C_{n}+\sum_{i=0}^{n-1}V_{P}\cdot C_{i}}{A}$$

where *V_R_* is the receptor compartment volume, *C_n_* is the drug concentration in the receptor at the time *n*, *V_P_* is the volume of the specimen removed, *C_i_* is the drug concentration in the receptor at the time *n*, *A* is the permeation area (cm^2^).

Permeation data are reported as the quantity of permeated naringin per permeation area related to time. All the permeation tests were conducted in triplicate; results are expressed as mean ± standard deviation.

### 2.8. Viability and Proliferation Assay on NuLi-1 Cell Line

Cell viability was analyzed using the MTT assay. Briefly, cells were seeded at the density of 10 × 10^3^/well, left to adhere to the plate and then treated with raw naringin and SAE formulations for 24 h. 3-(4,5-Methylthiazol-2-yl)-2,5-diphenyl-tetrazolium bromide (MTT) was added (0.5 mg/ml final concentration) to each well of the 96-well plate and incubated in 37 °C for 4 h. Formazan products were solubilized with 10% Triton X-100, 0.1 N HCl in 2-propanol. Absorbance was determined at 595 nm using a microplate reader (Bio-Rad Labaoratories srl, Milan, Italy) [51].

Cell growth was assessed using a colorimetric bromodeoxyuridine (BrdU) cell proliferation ELISA kit (Roche Diagnostics, Milan, Italy) and performed using a human airway epithelial cell line (NuLi-1). The NuLi-1 cell line was purchased from American Type Culture Collection (ATCC, Manassas, VA, USA). Briefly, 5 × 10^3^ cells were seeded into each coated well of a 96-well plate and left to adhere to the plate. The cells were then treated with increasing concentrations (from 15 to 100 μM) of naringin raw material or SAE naringin particles for 24 h, and bromodeoxyuridine was added for the final 16 h (10^−5^ mol/L). At the end of the whole cell culture period, the medium was removed, and the ELISA immunoassay was performed as described by the manufacturer. The colorimetric reaction was stopped by adding H_2_SO_4_, and the absorbance at 450 nm was measured using a microplate reader (Bio-Rad Laboratories, Milan, Italy). DMSO alone (0.1% final concentration in cell culture medium) did not yield any significant results in any biological in vitro assays.

### 2.9. Statistical Analyses

Experiments were independently repeated at least in triplicate. Error bars in the graphical data represent standard deviations. A Student’s t-test was used for statistical analysis, and statistical significance was claimed when the *p*-values were ≤0.05 (*), 0.01 (**) and 0.001 (***).

## 3. Results

### 3.1. Powder Preparation and Characterization

In all the supercritical experiments presented in this work, naringin was dissolved in ethanol at 2.0% (*w/v*) and precipitation temperature and pressure were varied. Two groups of experiments were performed: in the range of temperature (35–40 °C and at 60 °C.

The experiments performed at low temperature (35–40 °C) gave good results in terms of powder production, with a mass recovery of 70–95%, except the experiment performed at 40 °C, 80 bar, in which a large part of the product (80%) was extracted by the mixture SCCO_2_-ethanol and was found dissolved in the ethanol in the separator. In this particular condition the mass recovery is often poor or extremely low due to a mass loss in the downstream current continuously flowing out of the reactor, as well as to complex vapor-liquid mixture behaviour [52]. The operating conditions are reported in Table 1.

**Figure 2:**
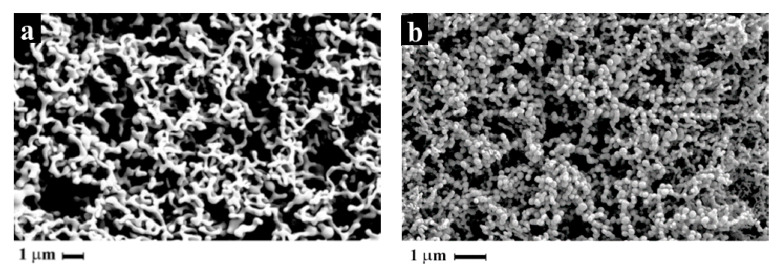
SEM photomicrographs of powder obtained at: (**a**) 80 bar, 40 °C (SAE#1); (**b**) 150 bar, 35 °C (SAE#3).

SEM analyses showed that at 40 °C, 80 and 100 bar connected particles were produced (Figure 2a); at 35 °C, 150 bar separated nanoparticles were produced (Figure 2b).

The PSD, reported in Table 2, showed that, in all the experiments, large diameters were measured, confirming the formation of aggregates during the suspension prepared for the analysis. The particles of the experiment performed at 35 °C and 150 bar showed smaller diameters, perhaps because there was no formation of strong aggregates during the precipitation. The size and the shape of the particles and the presence of aggregates have an influence on the breathability of the powder: the best results were obtained for the powder formed by separated particles (SAE#1 and SAE#4), whereas aggregates formed by nanoparticles (SAE#2 and SAE#3) were less capable of being broken during the flight. Evidently, by increasing the CO_2_ flow rate and the CO_2_ molar fraction, the formation of aggregates was strongly reduced. This also occurred when the powder was suspended in a liquid for aerosol target use because a better drying of ethanol was ensured during the process.

**Figure 3:**
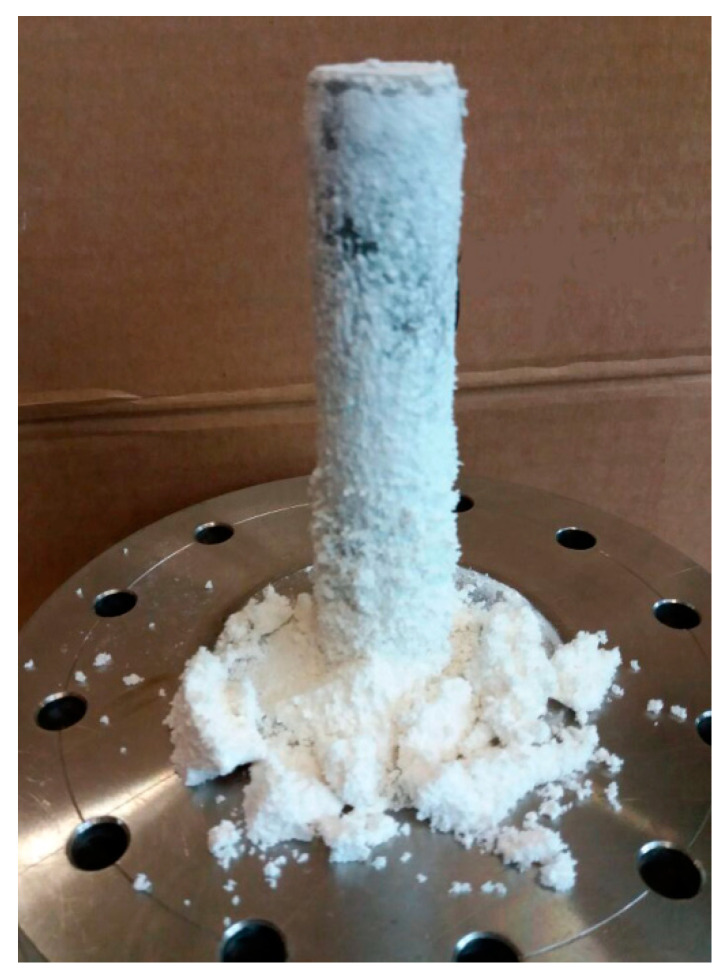
Micronized naringin collected on the apparatus filter.

The experiments performed at 60 °C showed high naringin production (an example of powder collected on the filter is reported in Figure 3), except when lower concentration of naringin in the starting solution was used (1% *wt*/*v*, SAE#10). All the operating conditions are reported in Table 3. In this case, the greatest portion of the naringin (about 90%) was found dissolved in the ethanol collected in the separator and the powder obtained was not enough for the analyses of PSD and breathability.

**Figure 4:**
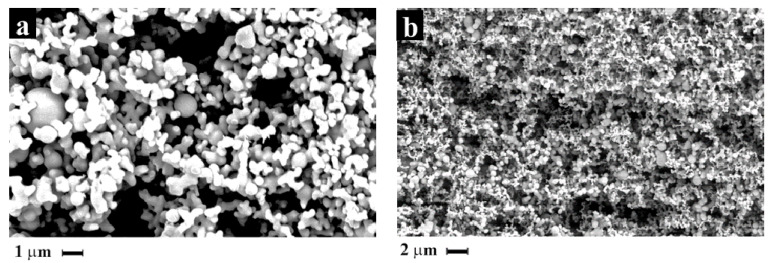
SEM photomicrographs of powder obtained at: (**a**) 110 bar, 60 °C (SAE#6); (**b**) 130 bar, 60 °C (SAE#8).

In all the experiments, spherical particles were obtained, and the degree of aggregation decreased with the increase in operating pressure, as shown by the SEM photomicrographs reported in Figure 4.

The PSD showed a smaller diameter size compared to the particles obtained at low temperature (Table 4), with the best results of mean diameter of 2.31 µm at higher pressure, corresponding to operating conditions far from the two-phase region. However, the best results in terms of breathability were obtained with the particles produced at a higher CO_2_ molar fraction (xCO_2_ = 0.99), with a mean diameter of 7.08 μm, perhaps because fewer aggregates were formed.

The analyses of the density of the powders, obtained as a direct value from the automatic gas ultra-pycnometer, showed that the particles obtained at 40 °C had a lower density (0.72–1.25 g/cm^3^) compared to those obtained at 60 °C (1.41–1.70 g/cm^3^).

**Figure 5:**
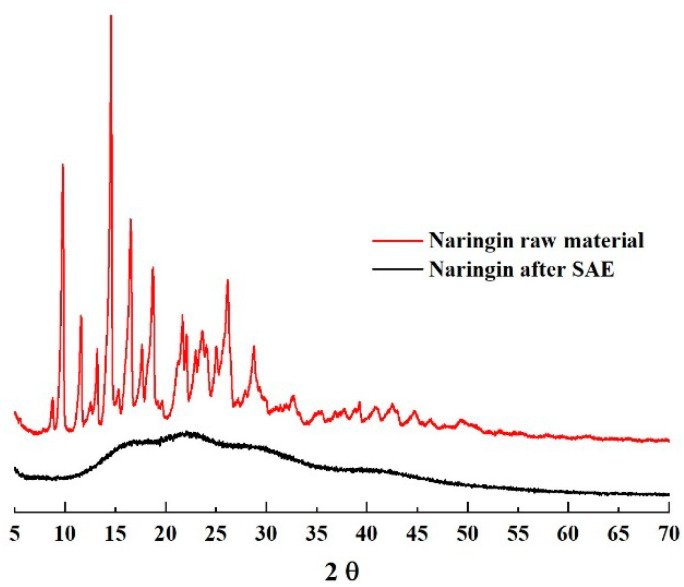
X-ray diffraction patterns of naringin as raw material and processed by SAE. SAE#9 has been reported as representative of all SAE-processed naringin formulations.

XRPD analyses of the raw naringin powder showed the presence of the characteristic peaks of the compound (see Figure 5), indicating that the powder is crystalline, whereas naringin microparticles produced by SAE showed the characteristic halo typical of an amorphous material. This is mainly correlated to the particle formation mechanism: in supercritical or nearly critical conditions, the surface tension of the system CO_2_-ethanol is close to zero, leading to a very fast nucleation and growth during particle formation, in turn resulting in a disordered molecular organization [53,54,55].

### 3.2. In Vitro Drug Permation Studies

**Figure 6:**
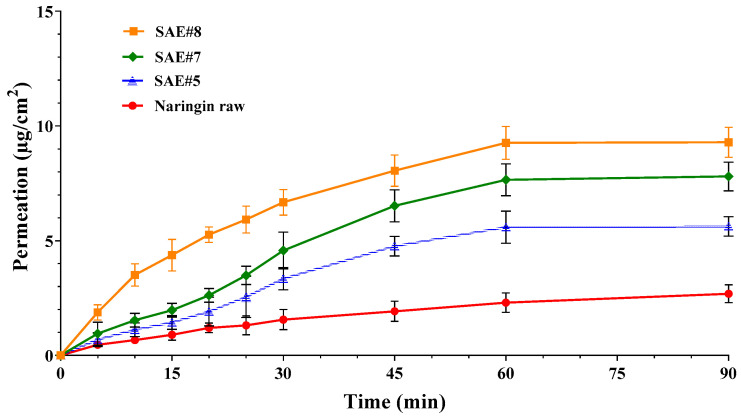
Permeation profiles of naringin formulations manufactured by SAE in comparison with raw material. Mean ± SD; (*n* = 6).

Bioavailability potential increase of the naringin processed by SAE was evaluated by permeation studies using Franz type vertical diffusion cells. In Figure 6 are reported the permeation profiles of naringin crystalline raw material, in comparison to three SAE formulations, SAE#5, SAE#7 and SAE#8. As expected, naringin raw material was able to permeate through the sintetic membrane very low over time (about 1.5 µg/cm^2^ at 30 min and 2.5 µg/cm^2^ after 90 min), mainly due to its low solubility (1.1 g/L). SAE formulations demonstrated higher permeability through membrane, up to 3.6-fold higher, this might be related to the reduction in particle size and the amorphization of the naringin, both contributing to enhance instant solubility, then permeability of the active biomolecule. In fact, SAE#5, the formulation with the highest FPF, demonstrated good permeation over time (about 3.3 µg/cm^2^ at 30 min and 5.8 µg/cm^2^ after 90 min) resulting more than 2-fold higher than pure naringin, while SAE#8, the formulation with the smallest particle size, exhibited the fastest permeation, (about 6.6 µg/cm^2^ at 30 min and 9.3 µg/cm^2^ after 90 min) resulting more than 3.5-fold higher than pure naringin and 1.5-fold faster than SAE#5. This might also be related to the shape of the particles, indeed the improvement of the permeation results is observed in correspondence of the increase of the operating pressure during particle formation, therefore to the decrease of the degree aggregation (Figure 4). In all cases, permeation declined after 45 minutes, reaching plateau values in about 1 h, probably due to the crystallisation of the amorphous naringin powders on the membrane surface [56].

**Figure 7:**
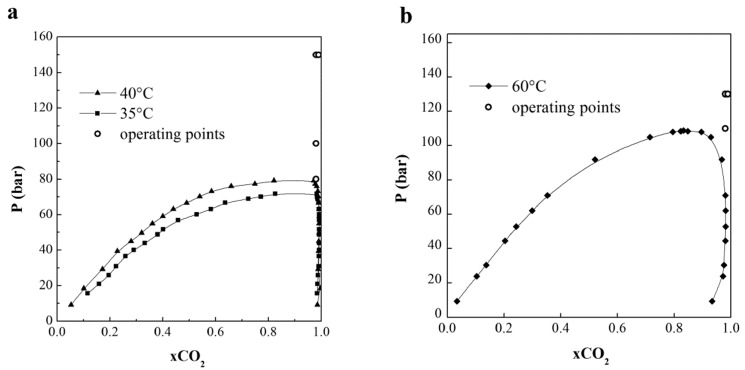
CO_2_ VLEs (**a**) adapted from Chang et al. [57] at 35 and 40 °C, and (**b**) from Jennings et al. [58] at 60 °C. The operating points of the experiments are also reported.

The obtained results can be also discussed considering the thermodynamic conditions during the particle formation, which strongly affect the properties of the obtained powder. The vapor liquid equilibria (VLE) of ethanol-CO_2_ and the operating conditions (drawn as the operating point) of both groups of experiments are reported in Figure 7 as a pressure vs. molar fraction diagram adapted from the literature The eventual influence of the presence of naringin on the equilibria is neglected, although it has been several times observed that the interaction of the solute with the solvent modifies the VLE for the system ethanol-CO_2_ [57]. From previous studies on several systems, it has been demonstrated that precipitation of separated particles can be obtained when the operating points are outside the two-phase region, though inside the two-phase region precipitation may occur from liquid phase and crystals or connected irregular particles are obtained [58,59].

This behavior was also observed in this work for naringin particle formation. At process conditions of 40 °C, and 80 and 100 bar, the operating point was close to the two-phase region (Figure 6) and connected particles were produced (Figure 2a); at process conditions of 35 °C, 150 bar and 60 °C, 110–130 bar, corresponding to the homogenous phase in which ethanol and CO_2_ are at supercritical conditions, separated nanoparticles were produced (Figure 2b and Figure 4).

Nanoparticles have the tendency to aggregate and deposit on the throat or to be blown out during expiration. The aggregation can be related to the electrostatic forces that become stronger with the decrease in the particle size or to the formation of hydrogen bonds. The latter can take place if there is even a small presence of solvent or humidity on the particle surface.

In the case of naringin particles obtained at low temperature, it was possible from the PSD to observe the formation of aggregates that resulted in a low fraction of naringin deposited on the lungs. When higher x_CO2_ was used, an improvement in deposition percentage was observed. At high temperatures, fewer aggregates were formed, but the particles were too small to deposit in the lungs and were lost during expiration, as could be observed by the PSD data. The highest percentage of deposited particles was obtained for the particles with D_50_~7 µm.

### 3.3. In Vitro Cytotoxicity

**Figure 8:**
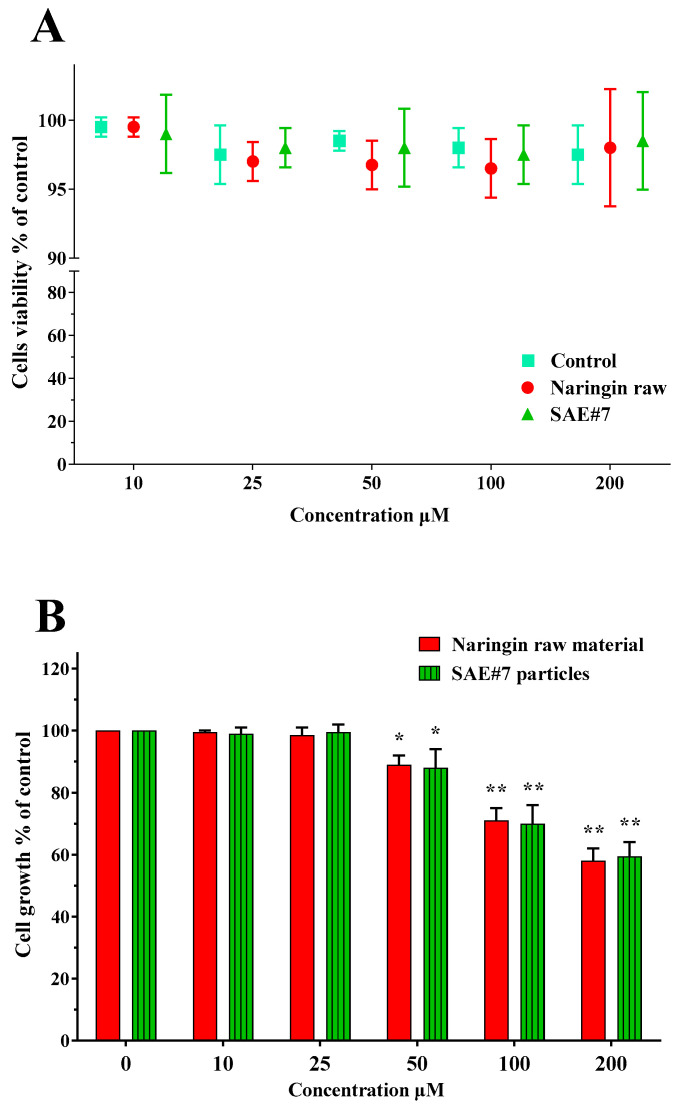
NuLi-1 cells treated for 24 h with raw naringin and SAE microparticles (SAE#7) at concentrations from 10 to 200 µM. Panel (**A**): Cell viability was determined by MTT assay. All data are shown as mean ± SD of three independent experiments, each performed in duplicate. Panel (**B**): Cell growth determined using a colorimetric bromodeoxyuridine cell proliferation ELISA kit. The histograms report the percentage of growing cells in comparison with untreated cells (control, 100% proliferation). All data are shown as mean ± SD of three independent experiments, each performed in duplicate (* *p* < 0.05 vs. control, ** *p* < 0.01 vs. control).

In order to determine any difference in cytotoxicity between naringin as raw material and SAE microparticles, immortalized bronchial epithelial NuLi-1 cells were used. Preliminary studies were performed to determine the cytotoxic concentration of naringin on the NuLi-1 cell line. After treatment of 24 h, unprocessed naringin did not show any significant effect on cell viability, as determined by MTT assay in the concentration ranging from 10 to 200 μM (see Figure 8A), but caused a dose-dependent reduction in cell growth. In fact, both naringin raw material and SAE#7 powder showed a reduction in cell growth from 50 to 200 μM in bronchial NuLi-1 cells, with a reduction of about 37% at the highest dose.

## 4. Conclusions

The supercritical antisolvent technique was successfully used to produce inhalable microparticles made of pure naringin without introducing any other excipient into the processed feed.

Very highly breathable microparticles, having an FPF over 32%, were obtained at operating conditions of T = 35 °C, P = 150 bar, xCO_2_ = 0.99. Increasing the operating temperature to 60 °C, while consequently reducing the pressure, led to the production of naringin powders having a higher FPF, of about 37%, due to a decrease in the particle size. Permeability of pure naringin particles obtained by SAE was significantly higher compared to that of naringin raw material, by up to 3.6-fold, due to both a reduction in the particle size and amorphization of the flavonoid. Such results demonstrate that the proposed technology can produce formulations with enhanced bioavailability and improved performance at the site of action after administration, with operating conditions of the atomization process can produce formulations able to both enhance bioavailability of the flavonoid and deliver a suitable concentration at the site of action after administration as well as maintaining good local concentration for an ex-tended period. Moreover, SAE naringin particles are not cytotoxic for bronchial epithelial cells at concentrations lower than 25 μM. The particles with the obtained characteristics show promise as use in an anti-inflammatory formulation as support for several kind of disease treatments, including the COVID-19 syndrome.

## Figures and Tables

**Table 1:** Operating conditions of experiments, ρ = CO_2_ density, T = temperature, P = pressure, xCO_2_ = CO_2_ molar fraction, Q_sol_ = solution flow rate.

Sample Code	T [°C]	P [bar]	ρ [Kg/L]	Q_sol_ [mL/min]	xCO_2_
SAE#1	40	80	0.28	1.0	0.98
SAE#2		100	0.62	1.0	0.98
SAE#3	35	150	0.81	1.0	0.98
SAE#4		150	0.81	0.5	0.99

**Table 2:** PSD and breathability of particles produced by supercritical antisolvent technique.

Sample Code	d_10_ (μm)	d_50_ (μm)	d_90_ (μm)	Dv Mean (μm)	Dose Loaded (mg)	Dose Emitted (%)	FPF (%)
SAE#1	3.31	9.78	17.64	10.38	5.5	97.2 ± 4.2	17.90
SAE#2	3.95	9.55	19.73	11.57	20	93.9 ± 5.9	4.04
SAE#3	5.63	12.60	24.87	14.00	15	94.5 ± 8.6	6.71
SAE#4	2.37	6.80	12.16	7.08	9	96.0 ± 2.1	32.60

**Table 3:** Operating conditions of experiments at T = 60 °C, ρ = CO_2_ density, P = pressure, xCO_2_ = CO_2_ molar fraction, Q_sol_ = solution flow rate. * Concentration naringin 1% *wt*/*v*.

Sample Code	P [bar]	ρ [Kg/L]	Q_sol_ [mL/min]	xCO_2_
SAE#5	90	0.24	1	0.98
SAE#6	110	0.36	1	0.98
SAE#7	110	0.36	0.5	0.99
SAE#8	130	0.50	1	0.98
SAE#9	130	0.50	0.5	0.99
SAE#10 *	130	0.50	0.5	0.99

**Table 4:** PSD and breathability of particles produced by the supercritical antisolvent technique.

Sample Code	d_10_ (μm)	d_50_ (μm)	d_90_ (μm)	Dv_mean_ (μm)	Dose Loaded (mg)	Dose Emitted (%)	FPF. (%)
SAE#5	1.975	7.087	19.220	7.21	12	93.8 ± 6.2	29.1 ± 2.1
SAE#6	1.233	6.553	15.28	7.98	12	96.6 ± 5. 9	22.7 ± 3.1
SAE#7	2.477	6.819	22.640	7.03	12	95.7 ± 8.6	23.4 ± 4.2
SAE#8	0.692	1.806	4.74	2.31	11	105 ± 10.9	27.8 ± 2.9
SAE#9	2.249	6.835	12.17	7.08	16	96.6 ± 3.3	36.8 ± 2.2

## Data Availability

Not applicable.

## References

[1] Carocho M., Ferreira I.C.F.R. (2013). A review on antioxidants, prooxidants and related controversy: Natural and synthetic compounds, screening and analysis methodologies and future perspectives. Food Chem. Toxicol..

[2] Del Gaudio P., Russo P., Dorado R.R., Sansone F., Mencherini T., Gasparri F., Aquino R.P. (2017). Submicrometric hypromellose acetate succinate particles as carrier for soy isoflavones extract with improved skin penetration performance. Carbohydr. Polym..

[3] Khan R.S., Grigor J., Winger R., Win A. (2013). Functional food product development—Opportunities and challenges for food manufacturers. Trends Food Sci. Technol..

[4] Nijveldt R.J., van Nood E., van Hoorn D.E.C., Boelens P.G., van Norren K., van Leeuwen P.A.M. (2001). Flavonoids: A review of probable mechanisms of action and potential applications. Am. J. Clin. Nutr..

[5] Rahman I., Biswas S.K., Kode A. (2006). Oxidant and antioxidant balance in the airways and airway diseases. Eur. J. Pharmacol..

[6] Liang Z., Ni R., Zhou J., Mao S. (2015). Recent advances in controlled pulmonary drug delivery. Drug Discov. Today.

[7] Buhl R., Meyer A., Vogelmeier C. (1996). Oxidant-protease interaction in the lung: Prospects for antioxidant therapy. Chest.

[8] Blomberg A. (2000). Airway inflammatory and antioxidant responses to oxidative and particulate air pollutants—experimental exposure studies in humans. Clin. Exp. Allergy.

[9] Fokkens W.J., Scheeren R.A. (2000). Upper airway defence mechanisms. Paediatr. Respir. Rev..

[10] Guo R.F., Ward P.A. (2007). Role of oxidants in lung injury during sepsis. Antioxid. Redox Signal..

[11] Schwarz K.B. (1996). Oxidative stress during viral infection: A review. Free Radic. Biol. Med..

[12] Auriemma G., Russo P., Del Gaudio P., García-González C.A., Landín M., Aquino R.P. (2020). Technologies and Formulation Design of Polysaccharide-Based Hydrogels for Drug Delivery. Molecules.

[13] Martín L., González-Coloma A., Adami R., Scognamiglio M., Reverchon E., Della Porta G., Urieta J., Mainar A. (2011). Supercritical antisolvent fractionation of ryanodol from Persea indica. J. Supercrit. Fluids.

[14] Oronsky B., Larson C., Hammond T.C., Oronsky A., Kesari S., Lybeck M., Reid T.R. (2021). A Review of Persistent Post-COVID Syndrome (PPCS). Clin. Rev. Allergy Immunol..

[15] Manniello M.D., Del Gaudio P., Porta A., Aquino R.P., Russo P. (2016). Aerodynamic properties, solubility and in vitro antibacterial efficacy of dry powders prepared by spray drying: Clarithromycin versus its hydrochloride salt. Eur. J. Pharm. Biopharm..

[16] Ruggiero V., Aquino R.P., Del Gaudio P., Campiglia P., Russo P. (2022). Post-COVID Syndrome: The Research Progress in the Treatment of Pulmonary sequelae after COVID-19 Infection. Pharmaceutics.

[17] Sansone F.M.T., Picerno P., Esposito T., Del Gaudio P., Russo P., Pepe G., Lauro M.R., Aquino R.P. (2014). Microencapsulation by spray drying of Lannea microcarpa extract: Technological characteristics and antioxidant activity. J. Pharm. Pharmacogn. Res..

[18] Del Gaudio P., Sansone F., Mencherini T., De Cicco F., Russo P., Aquino R.P. (2017). Nanospray drying as a novel tool to improve technological properties of soy isoflavone extracts. Planta Med..

[19] Manniello M.D., Del Gaudio P., Aquino R.P., Russo P. (2017). Clarithromycin and N-acetylcysteine co-spray-dried powders for pulmonary drug delivery: A focus on drug solubility. Int. J. Pharm..

[20] Telko M.J., Hickey A.J. (2005). Dry Powder Inhaler Formulation. Respir. Care.

[21] Chow A.H.L., Tong H.H.Y., Chattopadhyay T.P., Shekunov B.Y. (2007). Particle engineering for pulmonary drug delivery. Pharm. Res..

[22] Gilani K., Najafabadi A.R., Barghi M., Rafiee-Tehrani M. (2005). The effect of water to ethanol feed ratio on physical properties and aerosolization behavior of spray dried cromolyn sodium particles. J. Pharm. Sci..

[23] Loira-Pastoriza C., Todoroff J., Vanbever R. (2014). Delivery strategies for sustained drug release in the lungs. Adv. Drug Deliv. Rev..

[24] Chen R., Qi Q.-L., Wang M.-T., Li Q.-Y. (2016). Therapeutic potential of naringin: An overview. Pharm. Biol..

[25] John A.M., Najla G., Karel G. (2001). Biological Properties of Citrus Flavonoids Pertaining to Cancer and Inflammation. Curr. Med. Chem..

[26] Fakhri S., Piri S., Majnooni M.B., Farzaei M.H., Echeverria J. (2021). Targeting Neurological Manifestations of Coronaviruses by Candidate Phytochemicals: A Mechanistic Approach. Front. Pharmacol..

[27] Kumar S., Paul P., Yadav P., Kaul R., Maitra S.S., Jha S.K., Chaari A. (2022). A multi-targeted approach to identify potential flavonoids against three targets in the SARS-CoV-2 life cycle. Comput. Biol. Med..

[28] Liu W., Zheng W., Cheng L., Li M., Huang J., Bao S., Xu Q., Ma Z. (2022). Citrus fruits are rich in flavonoids for immunoregulation and potential targeting ACE2. Nat. Prod. Bioprospect..

[29] Sansone F., Aquino R., Del Gaudio P., Colombo P., Russo P. (2009). Physical characteristics and aerosol performance of naringin dry powders for pulmonary delivery prepared by spray-drying. Eur. J. Pharm. Biopharm..

[30] Rossmann M., Braeuer A., Schluecker E. (2014). Supercritical antisolvent micronization of PVP and ibuprofen sodium towards tailored solid dispersions. J. Supercrit. Fluids.

[31] Adami R., di Capua A., Reverchon E. (2017). Supercritical Assisted Atomization for the production of curcumin-biopolymer microspheres. Powder Technol..

[32] Hu D., Lin C., Liu L., Li S., Zhao Y. (2012). Preparation, characterization, and in vitro release investigation of lutein/zein nanoparticles via solution enhanced dispersion by supercritical fluids. J. Food Eng..

[33] Montes A., Gordillo M.D., Pereyra C., Santos D.D.L., De La Ossa E.M. (2014). Ibuprofen–polymer precipitation using supercritical CO2 at low temperature. J. Supercrit. Fluids.

[34] Prosapio V., De Marco I., Scognamiglio M., Reverchon E. (2015). Folic acid-PVP nanostructured composite microparticles by supercritical antisolvent precipitation. Chem. Eng. J..

[35] Wang W., Liu G., Wu J., Jiang Y. (2013). Co-precipitation of 10-hydroxycamptothecin and poly (l-lactic acid) by supercritical CO_2_ anti-solvent process using dichloromethane/ethanol co-solvent. J. Supercrit. Fluids.

[36] Adami R., Reverchon E., Järvenpää E., Huopalahti R. (2008). Supercritical AntiSolvent micronization of nalmefene HCl on laboratory and pilot scale. Powder Technol..

[37] Kim Y.H., Shing K.S. (2008). Supercritical fluid-micronized ipratropium bromide for pulmonary drug delivery. Powder Technol..

[38] Patomchaiviwat V., Paeratakul O., Kulvanich P. (2008). Formation of inhalable rifampicin-poly(l-lactide) microparticles by supercritical anti-solvent process. AAPS Pharmscitech.

[39] Reverchon E., Adami R., Caputo G. (2007). Production of Cromolyn Sodium Microparticles for Aerosol Delivery by Supercritical Assisted Atomization. AAPS Pharmscitech.

[40] Reverchon E., Della Porta G., Pallado P. (2001). Supercritical antisolvent precipitation of salbutamol microparticles. Powder Technol..

[41] Reverchon E., Adami R., Scognamiglio M., Fortunato G., Della Porta G. (2010). Beclomethasone Microparticles for Wet Inhalation, Produced by Supercritical Assisted Atomization. Ind. Eng. Chem. Res..

[42] De Paz E., Martin A., Every H., Cocero M.J., Alonso M.J.C. (2015). Production of water-soluble quercetin formulations by antisolvent precipitation and supercritical drying. J. Supercrit. Fluids.

[43] Kurniawansyah F., Mammucari R., Foster N. (2015). Inhalable curcumin formulations by supercritical technology. Powder Technol..

[44] Baldino L., Della Porta G., Osseo L.S., Reverchon E., Adami R. (2018). Concentrated oleuropein powder from olive leaves using alcoholic extraction and supercritical CO2 assisted extraction. J. Supercrit. Fluids.

[45] Chinnarasu C., Montes A., Ponce M.F., Casas L., Mantell C., Pereyra C., de la Ossa E.M. (2015). Precipitation of antioxidant fine particles from Olea europaea leaves using supercritical antisolvent process. J. Supercrit. Fluids.

[46] Garcí-Abarrio S., Marqués J., Scognamiglio M., Della Porta G., Reverchon E., Mainar A., Urieta J. (2012). Supercritical extraction and separation of antioxidants from residues of the wine industry. Procedia Eng..

[47] Meneses M.A., Caputo G., Scognamiglio M., Reverchon E., Adami R. (2015). Antioxidant phenolic compounds recovery from Mangifera indica L. by-products by supercritical antisolvent extraction. J. Food Eng..

[48] Natolino A., Da Porto C., Rodríguez-Rojo S., Moreno T., Cocero M.J. (2016). Supercritical antisolvent precipitation of polyphenols from grape marc extract. J. Supercrit. Fluids.

[49] Sosa M., Rodríguez-Rojo S., Mattea F., Cismondi M., Cocero M. (2011). Green tea encapsulation by means of high pressure antisolvent coprecipitation. J. Supercrit. Fluids.

[50] Visentin A., Rodríguez-Rojo S., Navarrete A., Maestri D., Cocero M. (2012). Precipitation and encapsulation of rosemary antioxidants by supercritical antisolvent process. J. Food Eng..

[51] Palazzo I., Lamparelli E.P., Ciardulli M.C., Scala P., Reverchon E., Forsyth N., Maffulli N., Santoro A., Della Porta G. (2021). Supercritical emulsion extraction fabricated PLA/PLGA micro/nano carriers for growth factor delivery: Release profiles and cytotoxicity. Int. J. Pharm..

[52] Gimenez-Rota C., Palazzo I., Scognamiglio M.R., Mainar A., Reverchon E., Della Porta G. (2019). β-Carotene, α-tocoferol and rosmarinic acid encapsulated within PLA/PLGA microcarriers by supercritical emulsion extraction: Encapsulation efficiency, drugs shelf-life and antioxidant activity. J. Supercrit. Fluids.

[53] Adami R., Osseo L.S., Huopalahti R., Reverchon E. (2007). Supercritical AntiSolvent micronization of PVA by semi-continuous and batch processing. J. Supercrit. Fluids.

[54] Araus K.A., Casado V., del Valle J.M., Robert P.S., de la Fuente J.C. (2019). Cosolvent effect of ethanol on the solubility of lutein in supercritical carbon dioxide. J. Supercrit. Fluids.

[55] Della Porta G., Campardelli R., Reverchon E. (2013). Monodisperse biopolymer nanoparticles by Continuous Supercritical Emulsion Extraction. J. Supercrit. Fluids.

[56] Sansone F., Picerno P., Mencherini T., Russo P., Gasparri F., Giannini V., Lauro M.R., Puglisi G., Aquino R.P. (2013). Enhanced technological and permeation properties of a microencapsulated soy isoflavones extract. J. Food Eng..

[57] Chang C.J., Day C.Y., Ko C.M., Chiu K.L. (1997). Densities and P-x-y diagrams for carbon dioxide dissolution in methanol, ethanol, and acetone mixtures. Fluid Phase Equilibr.

[58] Jennings D.W., Lee R.J., Teja A.S. (1991). Vapor-liquid equilibria in the carbon dioxide + ethanol and carbon dioxide + 1-butanol systems. J. Chem. Eng. Data.

[59] Campardelli R., Della Porta G., Reverchon E. (2012). Solvent elimination from polymer nanoparticle suspensions by continuous supercritical extraction. J. Supercrit. Fluids.

